# Improving Patient Access to New Treatment Modalities in European Cancer Care

**DOI:** 10.3390/jmahp14040061

**Published:** 2026-10-16

**Authors:** Christian K. Schneider, Hemal Morjaria, Sacha Wissink, Christophe M. Deroose, Vladimir Galvao, Viktor Grünwald, Oliver C. Kiß, Stefan N. Symeonides

**Affiliations:** 1 Cencora PharmaLex, Basler Straße 7, 61352 Bad Homburg vor der Höhe, Germany; 2 TWINCORE Centre for Experimental and Clinical Infection Research, Feodor-Lynen-Str. 7, 30625 Hannover, Germany; 3 Cancer Drug Development Forum (CDDF), Clos Chapelle-aux-Champs 30, 1200 Woluwe Saint Lambert, Belgium; 4 Global Regulatory Affairs Oncology, Johnson & Johnson Innovative Medicine, 50-100 Holmersfram Way, High Wycombe HP12 4DP, UK; 5 Regulatory Affairs Europe, MSD, Molenstraat 110, 5342 CC Oss, The Netherlands; 6 Nuclear Medicine and Molecular Imaging, Department of Imaging and Pathology, KU Leuven, Herestraat 49, 3000 Leuven, Belgium; 7 Research Unit for Molecular Therapy of Cancer (UITM)—CaixaResearch, CELLEX CENTER, Natzaret, 115-117, 08035 Barcelona, Spain; 8 Carolus Institute for Urologic Oncology, University Hospital Essen, Hufelandstraße 55, 45147 Essen, Germany; 9 Institute of Radiopharmaceutical Cancer Research, Helmholtz-Zentrum Dresden-Rossendorf (HZDR), Bautzner Landstrasse 400, 01328 Dresden, Germany; 10 Edinburgh Experimental Cancer Medicine Centre, Edinburgh Cancer Centre, NHS Lothian, Institute of Genetics and Cancer, University of Edinburgh, Crew Road, Edinburgh EH4 2XU, UK; 11 Cancer Research UK (CRUK) Scotland Centre, Institute of Genetics and Cancer, University of Edinburgh, Crew Road, Edinburgh EH4 2XU, UK

**Keywords:** targeted oncology, patient access, regulatory frameworks, radioligand therapy, cellular therapies, health policy, healthcare infrastructure, interdisciplinary collaboration, health technology assessment (HTA)

## Abstract

Although new cancer treatment modalities such as cellular therapies and radiopharmaceuticals have proven to be potentially transformative treatment options, there remain shortcomings with their equitable implementation in routine clinical practice across Europe, the patient-centricity of their associated patient pathways, and their hidden costs. Patient access can only be achieved if the necessary healthcare infrastructure is in place to manage patient pathways and manufacturing can be faithfully scaled from clinical trial to routine clinical practice. This multi-stakeholder perspective highlights practical challenges and solutions to bridge these gaps, based on the multi-stakeholder Cancer Drug Development Forum (CDDF) Workshop ‘New modalities in cancer drug development’ (Brussels, 31 March–1 April 2025), sharing insights from academics, regulators, patient groups, industry, and non-commercial organisations. It is essential to anticipate any healthcare deployment considerations if new treatment modalities are to achieve their full potential in terms of patient experience. Earlier open dialogue with regulatory bodies, payers and healthcare providers can help prepare for the practicalities of their national roll-out. These multi-stakeholder discussions provided actionable recommendations for policymakers, regulators, and healthcare providers to address barriers in deploying novel cancer therapies. A concerted effort from all stakeholders is needed to improve the legislative and infrastructural environments needed to expedite effective patient access to these novel targeted oncology treatments.

## 1. Background—Lessons Learnt

Despite major scientific advancements in cancer treatment, challenges remain with rolling out new modalities in clinical practice, often with a lag between approval/reimbursement and routine use, ultimately hindering equitable patient access in already strained healthcare systems across Europe. Alongside its clinical development, it is important to envisage how a novel targeted oncology therapy will embed into existing healthcare systems and respective patient pathways. Many of the current bottlenecks in oncology drug development appear downstream of positive clinical data reporting, with hurdles ranging from the healthcare “macrospace” (e.g., outdated legislation slowing drug approval) to the “microspace” (e.g., insufficient dedicated hospital beds to receive treatment) (Table 1).

Regulatory frameworks and healthcare infrastructure can lag in their ability to accommodate the swift deployment of promising new treatment modalities, with marketing authorisation (MA) of new cancer therapies not necessarily translating to patient access [2].

Lessons have been learnt from earlier novel cancer treatment modalities, such as radioligand therapy (RLT) and CAR T-cell therapy (CAR-T). Although RLT is showing great promise in neuroendocrine tumours and prostate cancer (e.g., [3]), its implementation has also highlighted a lack of specialist resources in EU public healthcare systems for its delivery and manufacturing. Factors include the lack of availability/reimbursement of specialist positron emission tomography imaging in some European countries; availability of nuclear medicine inpatient beds in specialised centres and meeting legal requirements for radioprotection (varies across Europe); and sufficient trained nuclear medicine personnel (e.g., physicians, technologists, medical physicists, radiopharmacists) [4]. Furthermore, the patient experience can be complicated by the need to navigate between different disconnected disciplines (e.g., for prostate cancer: urology, oncology, and nuclear medicine departments). In addition to these infrastructural shortcomings, EU regulatory frameworks for radiopharmaceuticals are not sufficiently adapted to current needs and would benefit from further updates (Section 2).

Adoptive cellular therapies, such as CAR-T, have been available for the treatment of various haematological malignancies, with clinical research now showing promise in earlier treatment lines and also expanding to solid tumours [5]. CAR-T are among 27 Advanced Therapy Medicinal Products (ATMPs) approved by the European Medicines Agency (EMA) since 2009 (only 19 were actively approved at the time of writing due to withdrawal/unrenewed MA), of which around half are for cancer indications [6]. However, CAR-T delivery remains a very complex, individualised, and multi-step process (apheresis, manufacturing, lymphodepletion and treatment, and post-therapy) [7], with important considerations around safety and response monitoring due to the continued ongoing risk of cytopenias, immunosuppression, and infection post-CAR-T. Managing potentially life-threatening immune-related adverse effects following CAR T-cell infusion is crucial for optimal patient follow-up, aided by establishing a CAR-T shared care model with all referral centres including nurse-led ‘late toxicity’ clinics [8].

Although cellular therapy has proven to be a transformative treatment modality in targeted oncology over the last decade, it also requires close-to-bedside manufacturing. The hidden costs of the logistical complexities and manufacturing required to equitably and safely deliver this treatment model are now apparent, putting a strain on healthcare systems and sometimes halting the development of new cellular therapies [9,10]. In terms of regulatory framework, in addition to the normal legislation for medicinal products, ATMPs are subject to a targeted regulation created in 2007 (‘lex specialis’ Regulation (EC) No 1394/2007) with specific provisions to facilitate regulatory assessment including the introduction of the Committee for Advanced Therapies, mandatory use of the centralised procedure, and a risk-based approach supported by post-MA efficacy and safety follow-up requirements. Such a pre-emptive regulatory approach enabled the approval and governance of these novel therapies, serving as an important example of proactively adapting existing frameworks to bring new modalities to patients.

## 2. Regulatory Unmet Needs Remain Across Multiple Modalities

Unlike CAR-T, some new modalities like radiopharmaceuticals are not sufficiently covered by specific existing EU regulatory guidance and legislations, complicating their advance into the clinic [11]. Radiopharmaceuticals can be divided into three regulatory categories, each with their respective legislations, based on their use: MA, clinical trials, and extemporaneous or magistral preparations (compounding in-house). All radiopharmaceuticals considered to be medicinal products for human use require MA (except for investigational medicinal products and magistral and officinal formulae) and must be produced under Good Manufacturing Practice (GMP). However, the relevant EU Directive 2001/83/EC, developed during the 1980s–early 1990s and which came into force in 2001, was not written with modern theranostic radiopharmaceuticals in mind [12] and has not yet been updated in this respect. For example, the current legislation does not differentiate between a radionuclide precursor as a medicinal product in a kit versus as a starting material for in-house radiosynthesis; therefore, a clinical institution with a GMP-compliant radiopharmacy cannot legally purchase a radionuclide precursor as a starting material (lacking MA) from an off-site location to prepare a radiopharmaceutical in-house [13]. Hence, updating Directive 2001/83/EC to make this distinction would facilitate the deployment of novel radiopharmaceuticals [11].

The European Atomic Energy Community (EURATOM) legislation is important for regulating radiopharmaceutical use; however, it currently does not distinguish between external beam radiation treatment and radiopharmaceutical therapy, despite substantial differences in modes of administration, spatiotemporal control, dose homogeneity, and dose rate of the radiation. Specific regulation for therapeutic nuclear medicine (i.e., RLT), with an emphasis on quality and feasibility, would facilitate their transition from a clinical trial to clinical practice setting. Other important regulatory unmet needs in the radiopharmaceutical space include: greater consistency between EU countries regarding radioprotection (i.e., duration of inpatient stay, with specific emphasis on reducing mandatory stays) and for magistral preparations (currently highly variable with complex interpretation), with an emphasis on maintaining this valuable part of the radiopharmaceutical landscape, increasing patient access; creating a realistic framework for dosimetry throughout the EU, including incorporating appropriate dosimetry assessments during clinical development; and global regulatory harmonisation [14,15].

Among other personalised oncology therapies in development are therapeutic immunotherapies. Sipuleucel-T led the way with approval for prostate cancer (currently only available in the US; MA withdrawn in Europe for commercial reasons [16]), now followed by a wave of RNA-based vaccines with individualised neoantigen selection. Regulatory guidance has been complicated by lack of alignment between regulatory bodies regarding definitions around cancer vaccines (e.g., UK, EU versus US) [17,18] but remains in ongoing development. Furthermore, the EMA updated the Anti-Cancer Guideline for this novel class of product [19], and the UK Medicines and Healthcare products Regulatory Agency (MHRA) has recently published draft guidance [20].

## 3. Infrastructural Hurdles: Manufacturing, Quality-Check, Delivery, and Patient Pathways

Planning for post-approval evidence generation can clear the path for a smooth and speedy MA and future market access; however, this requires an appropriate healthcare infrastructure to promote robust evidence generation in routine practice. As with radiopharmaceuticals and cellular therapies, each new modality is associated with its individual infrastructural requirements, from manufacturing to its safe and effective delivery to patients, including specialised centres and treatment rooms, qualified and trained personnel, and a seamless healthcare structure from diagnosis, application, and follow-up. From the payers’ perspective, however, experience with cellular therapies has not always been cost-effective, due to lack of facilities for adequate treatment delivery (e.g., ≥ 10-day inpatient stay blocking out units) and unsustainable manufacturing. This could be managed through proportionate and balanced regulation of decentralised manufacturing consistently across the EU, per the recent UK initiative [21]; sound legislation on GMP for ATMPs adapted to local manufacturing (versus the more “standard” centralised manufacturing); and the expansion of cellular therapy delivery to local treatment centres. Moving forward, individual countries must work on national infrastructure hubs, decrease cost by learning from other products and previous cases (e.g., platform technology for manufacturing), and draw up best practice guidance.

## 4. Health Technology Assessment (HTA) and Payer Challenges

The increased need for specialised infrastructure required to support the routine implementation of these new targeted oncology treatment modalities adds to the overall cost—a consideration for health technology assessment evaluating their reimbursement by national healthcare systems alongside other factors such as cost to buy/manufacture; the potential incremental benefit vs. existing treatment(s); one-off therapy vs. multiple treatments; and whether sufficient infrastructure is already in place or if any added cost to governments is required to bridge the gap between technology and healthcare delivery. As a society, we must consider the added costs of making new, more complex treatment modalities available and manage our expectations regarding their real added value—e.g., some new targeted oncology modalities might not be breakthroughs like CAR-T but may just have a small, incremental effect, or benefit only specific patients, or become a valuable combination partner down the line. Improvements in healthcare structures and connectivity across disciplines will be key to promote cross-sectional patient pathways for more efficient use of public resources.

## 5. Conclusions

The clinical development of a new first-in-class modality involves reaching scientific/clinical milestones whilst navigating the available legislations and healthcare systems which may not be fit for the purpose if its make-up, production, and/or application is distinct from existing therapies. Working together as a community, how can we adapt our systems to fast-track the clinical delivery of innovative, evidence-based cancer therapies to patients? Early and consistent communications between all stakeholders to plan ahead for post-MA approval roll-out can ensure a smooth transition from the clinical trial setting (from which lessons can be learned on logistics and other practical measures) to implementation in routine clinical practice. Furthermore, adapting healthcare structures and connectivity across disciplines will be key to promote cross-sectional patient pathways (Box 1).

Box 1 **Measures to improve patient access to new and innovative cancer treatment modalities.**

- Better engagement between industry, academia, cancer societies, regulators, and payers to raise awareness of real-world practicalities ‘on the ground’ and flag relevant shortcomings in the healthcare system early on (i.e., regarding manufacturing, availability of specialised centres and resources, cost/feasibility, etc.).
  ○ For example, regional cancer societies/organisations could raise any foreseeable practical issues when updating treatment guidelines with a new modality and feedback to regulatory authorities in early decision-making.

- Creating a proportionate and balanced regulatory framework providing high quality, safety, and oversight on one hand, and “doability” by hospitals on the other.
  ○ As a community, if relevant legislations cannot be updated in the immediate term to meet the current state of technological advancement (e.g., radiopharmaceuticals), interim regulatory scientific guidance should be drawn up by a neutral forum of relevant stakeholders/experts (e.g., European Association of Nuclear Medicine [EANM]) to support routine delivery and contribute to new legislation.

- Bridge the infrastructural gaps in national healthcare systems by incentivising funding to accelerate the deployment of new cancer treatment modalities in routine clinical practice.
  ○ Public–private collaboration strategies, e.g., relevant industry stakeholders supporting that initial deployment and some of its upfront costs.
  ○ National and supra-national coordination, e.g., facilitating universal access to highly specialised resources via cross-border networks of clinical specialist centres, such as the European Reference Networks.

## Figures and Tables

**Table 1:** Hurdles to deploying new treatment modalities in oncology.

Aspects	Considerations/Examples
**Regulatory challenges for swift drug approvals**	Incremental benefit versus blockbuster expectations (“better than the Beatles” problem [1]) New modality may not fit with existing legal provisions Quality assurance for local manufacturing: re-evaluating traditional GMP principles to maintain quality in smaller, geographically dispersed sites
**Infrastructural challenges for application in routine clinical practice**	Standardised manufacturing and quality assurance procedures (e.g., nationally) and local implementation Resources/Training: How to get sufficient numbers of highly trained staff Establishing a routine long-term toxicity measurement system outside of formal registries
**Health economic challenges for HTA & payers for equitable patient access**	Readiness (or not) of existing healthcare systems to safely and routinely deploy a given new modality As the indications of new modalities expand, there is greater demand for specialised infrastructure, e.g., availability/access to specialised centres for patients in rural areas Cost-effectiveness modelling should integrate all factors to include specialist infrastructure and access for all patients

GMP, Good Manufacturing Practice; HTA, health technology assessment.

## Data Availability

No new data were created or analyzed in this study. Data sharing is not applicable to this article.
